# Analysis of genetic admixture in Uyghur using the 26 Y-STR loci system

**DOI:** 10.1038/srep19998

**Published:** 2016-02-04

**Authors:** Yingnan Bian, Suhua Zhang, Wei Zhou, Qi Zhao, Siqintuya, Ruxin Zhu, Zheng Wang, Yuzhen Gao, Jie Hong, Daru Lu, Chengtao Li

**Affiliations:** 1 Shanghai Key Laboratory of Forensic Medicine, Institute of Forensic Sciences, Ministry of Justice, P.R. China, Shanghai 200063, China; 2 State Key Laboratory of Genetic Engineering, Institute of Genetics, School of Life Sciences, Fudan University, Shanghai 200433, China; 3 University of Pennsylvania, 3451 Walnut Street, Philadelphia, PA 19104, United States; 4 Department of Forensic Medicine, Medical College of Soochow University, Suzhou 215123, China; 5 Shihezi City Public Security Bureau Traffic Institute of Science and Technology, Xinjiang 832000, China; 6 Division of Gastroenterology and Hepatology, Renji Hospital, Shanghai Institution of Digestive Disease; Key Laboratory of Gastroenterology and Hepatology, Ministry of Health; State Key Laboratory of Oncogene and Related Genes, Shanghai Jiao-Tong University School of Medicine, Shanghai 200001, China

## Abstract

The Uyghur population has experienced extensive interaction with European and Eastern Asian populations historically. A set of high-resolution genetic markers could be useful to infer the genetic relationships between the Uyghur population and European and Asian populations. In this study we typed 100 unrelated Uyghur males living in southern Xinjiang at 26 Y-STR loci. Using the high-resolution 26 Y-STR loci system, we investigated genetic and phylogenetic relationship between the Uyghur population and 23 reference European or Asian populations. We found that the Uyghur population exhibited a genetic admixture of Eastern Asian and European populations, and had a slightly closer relationship with the selected European populations than the Eastern Asian populations. We also demonstrated that the 26 Y-STR loci system was potentially useful in forensic sciences because it has a large power of discrimination and rarely exhibits common haplotypes. However, ancestry inference of Uyghur samples could be challenging due to the admixed nature of the population.

Uyghurs live primarily in Xinjiang, a province in the far western region of China and crossed by the Silk Road which is an important pathway connecting Eastern Asia with Central Asia and Europe. As a result, Uyghurs have experienced extensive interaction with other Asian and European populations. Modern Uyghurs present an admixture of Eastern and Western anthropological and genetic traits^1^,^2^,^3^. To shed light on the historical interactions of the Uyghurs with the Europeans and Eastern Asians, a high-resolution genetic dataset as well as detailed population genetics and phylogenetic analyses based on the dataset are needed. Such a high-resolution dataset is also potentially useful in forensic applications either within Uyghur populations or to infer ancestry of DNA donors.

Y chromosome contains the largest non-recombining block in human genome and can be used to trace the male line of descent^4^. Short tandem repeats (STRs) are genetic markers that are more informative than single nucleotide polymorphisms (SNPs) and reveal more recent events in population history, because of its high mutability and high degree of allelic polymorphism. A number of highly polymorphic Y chromosome STRs (Y-STRs) systems are useful and available for studies in population genetics and forensic sciences such as patrilineal relationship evaluation, mixture identification and ancestry inference^5^,^6^,^7^,^8^,^9^. Such Y-STRs systems have been successfully applied to Uyghur populations^10^,^11^,^12^,^13^.

In this study we studied the genetic diversity at 26 Y-STR loci of Uyghurs living in southern Xinjiang and used them to infer genetic relationships between the Uyghur population and different European and Asian populations. In addition, we presented and compared forensic parameters for different Y-STR systems and discussed the potential application of the Y-STR loci system to infer ancestry of DNA donors that are potentially from a admixed population, like Uyghur.

## Methods

### Samples used in the study

The following procedures were in accordance with the humane and ethical research principles and were approved by the Ethical Committee of Institute of Forensic Science, Ministry of Justice, China.

A total of 100 samples from unrelated Uyghur males recruited from southern Xinjiang were collected. Informed consent was obtained from all participants. For each individual, there was no consanguineous marriage or intermarriages with other ethnic groups within the latest three generations.

**Figure 1:**
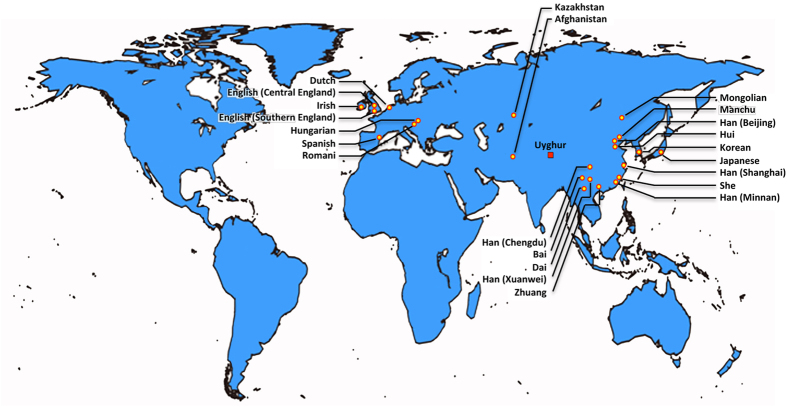
Geographic distribution of populations used in this study. 100 samples from unrelated Uyghur males living in southern Xinjiang were collected. 23 different populations in Europe, Central Asian and Eastern Asia containing a total of 7696 haplotypes were used as the reference populations. The map was created by package maps under R program (V3.02).

23 different populations in Eastern Asia, Central Asian and Europe containing a total of 7696 haplotypes were selected as reference populations (Table 1). The geographical locations of the reference populations were shown in Fig. 1.

### DNA extraction

Genomic DNAs were extracted from blood stains using the Chelex-100 method as described by Walsh *et al.*^14^. In brief, each bloodstain (approximately 3 mm × 3 mm) was incubated in 1 ml water for 30 minutes at room temperature before vortexed for 15 s and centrifuged for 3 minutes at 14,000 rpm. Supernatant was then removed and the pellet was incubated in 200 μl of 5% Chelex for 30 minutes at 56 °C. The mixture was boiled for 8 minutes and centrifuged for 3 minutes at 14,000 rpm. The supernatant, which contained the genomic DNAs, was aliquoted and stored at −20 °C.

### PCR amplification and Y-STR typing

26 Y-STR loci (DYS19, DYS389I, DYS389II, DYS390, DYS391, DYS392, DYS393, DYS385ab, DYS437, DYS438, DYS439, DYS448, DYS456, DYS458, DYS635, Y_GATA_H4, DYS576, DYS570, DYS481, DYS533, DYS549, DYS643, DYS460, DYS449 and DYS388) were amplified using the Goldeneye^®^ 26Y system (PEOPLESPOTINC R&D, China) as described in our previous study^15^. AGCU Database Y24 STR kit (AGCU ScienTech Incorporation, China) and AGCU GFS 24Y STR kit (AGCU ScienTech Incorporation, China) were used to confirm the null alleles, intermediate alleles and duplication variants according to the manufacturer’s protocol. The DNAs from 9947A and 9948 cell lines (Promega Corporation, USA) were used as negative and positive controls, respectively. The PCR products were separated and detected by capillary electrophoresis on an ABI 3130xL Genetic Analyzer (Applied Biosystems, USA). The genotyping results were analyzed using GeneMapper ID v3.2 (Applied Biosystems, USA).

### Statistical analyses

Allelic and haplotype frequencies were calculated by direct counting. Genetic diversity (GD) of single-marker was calculated using Nei’s formula GD = n (1-ΣP_*i*_^2^)/(n-1), where P_*i*_ is the relative frequency of the *i*-th allele and n is the sample size^16^,^17^. Haplotype diversity (HD) was calculated in an analogous way to GD through replacing the allele frequencies (P_*i*_) by the relative frequencies of different haplotypes. Haplotype discrimination capacity (DC) was calculated as the ratio of unique haplotypes in the sample. Match probabilities (MP) were calculated as ΣP_*i*_^2^, where P_*i*_ is the frequency of the *i*-th haplotype. To analyze genetic distances between the Uyghur population and the reference populations, the analysis of molecular variance (AMOVA) and multidimensional scaling (MDS) that maximizes variation among populations were performed using YHRD online tools (http://www.yhrd.org) based on pairwise *R*_ST_ values.

### Phylogenetic analysis

A neighbor-joining phylogenetic tree was constructed for the Uyghur and the reference populations based on a distance matrix of *R*_ST_ using the T-REX web server^18^.

Phylogenetic analysis was also carried out on haplogroup level with individual samples used in this study. Y-DNA haplogroup of each individual sample was predicted using the offline version of Vadim Urasin’s YPredictor (http://predictor.ydna.ru/). A Y-DNA haplogroup tree was adopted from International Society of Genetic Geneology to show the distribution of samples among haplogroups.

### Linear discriminant analysis

Linear discriminant analysis (LDA) was performed on Uyghur, European, Central Asian and Eastern Asian samples using XLSTAT (http://www.xlstat.com/en/). The multi-copy marker DYS389 and markers that have null alleles or duplication variants in multiple samples in the Uyghur population or any of the reference populations were excluded from the analysis, resulting the following markers used for the analysis: DYS393, DYS390, DYS439, DYS391, DYS392, DYS458, DYS437, DYS448, Y_GATA_H.

## Results and Discussion

### Genetic relationship between the Uyghur population and reference populations in Eastern Asia or Europe

**Figure 2:**
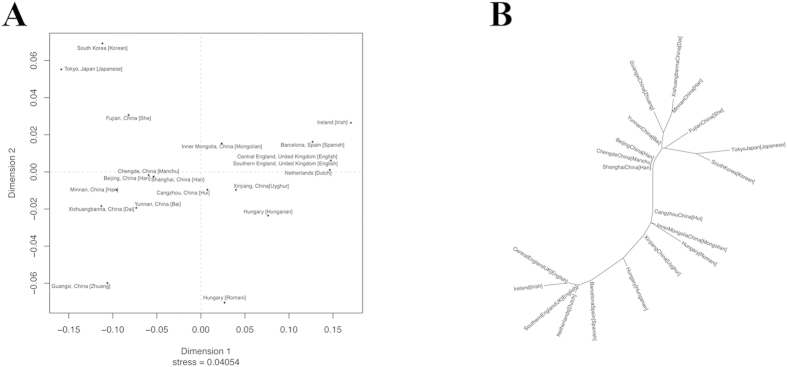
MDS plot and neighbour-joining phylogenetic tree. (**A**) MDS plot of the Uyghur population and 19 different European or Eastern Asian populations based on *R*_ST_. (**B**) Neighbour-joining phylogenetic tree of the Uyghur population and the European and Eastern Asian populations based on a distance matrix of *R*_ST_.

The detailed typing results at the 26 Y-STR loci of 100 male individuals of Uyghur from southern Xinjiang are shown in Supplementary Table S1. Uyghur is known to be an admixture of Eastern Asian and European populations^19^. Using the high-resolution Y-STR loci system, we studied the genetic relationship of Uyghur and different Asian or European populations (Table 1) based on *R*_ST_ (Table 2), and MDS was used to visualize the results (Fig. 2A). To avoid using populations exclusively from the Eastern or Western extremes of Eurasia continent, we also included samples from Kazakhstan and Afghanistan that are in Central Asia. As is shown by the MDS plot (Supplementary Figure S1), the Uyghur population lies between the Eastern Asian and European populations. Our results are consistent with the hypothesis that both Eastern Asian and European populations contributed to the current gene pool of the Uyghur population. Uyghur populations are also genetically close to Central Asian populations, reflecting the communications among the populations due to geographic proximity, silk roads and the genetic contribution of the Mongols suggested by previous studies^20^,^21^,^22^,^23^. On the other hand, Central Asian populations are also closely related to Eastern Asian and European populations, consistent with previous studies suggesting the admixed nature of Central Asia^21^,^22^,^23^,^24^,^25^. The two observations collectively substantiate the inference that Eastern and Western Eurasian populations are genetic donors of Uyghur and its closely related Central Asian populations. However, because the only Y-STR data available for Central Asian populations are based on 17 Y-STR loci, there is a higher chance to infer biased genetic distances based on the available data compared to using a dataset based on 23 Y-STR loci typed using the PowerPlex Y23 kit. Such bias may explain the unexpectedly long genetic distance from Han populations to the rest of the populations, and the clustering of several geographically and genetically isolated minorities in southern and northern China (Supplementary Figure S1). Among the 19 reference populations from Europe and Eastern Asia where information on 23 Y-STR loci is available, Hui is the most closely related to Uyghur (*R*_ST_ = 0.0132) and Dai is the most distantly related to Uyghur (*R*_ST_ = 0.1717). The AMOVA results also show that the Uyghur population is slightly more closely related to the European populations than the Eastern Asian populations (p = 0.074), which is consistent with the studies of Xu *et al.* using SNP markers^19^, Zhao *et al.* using classical markers^25^ and Comas *et al.* using mtDNAs^26^. However, the observed genetic relationships could be complicated by many factors including the geographic origins of the samples, the choice of genetic markers and the coverage of reference populations^27^. A number of other studies suggest that Uyghur is more closely related to Eastern Asian populations than European populations^27^,^28^,^29^. One possible cause of the discrepancy is the difference in the geographic locations of the Uyghur populations that are analyzed in different studies. The Uyghur populations described in the present study and in the study of Xu *et al.* are in southern Xinjiang, which are less affected by the recent migrations of Han Chinese^30^,^31^.

Evolutionary relationships between the Uyghur population and the Asian and European populations are inferred from the Neighbor-joining tree based on the *R*_ST_ values (Fig. 2B). It has been shown that, in neighbor-joining trees, an admixed population will always lie on the path between the source populations^32^. Indeed, the Uyghur population lies between the European populations and the Eastern Asian populations. The distance-based phylogeny is strongly supportive of the admixed nature of the Uyghur population and the Central Asian populations. Similar to the MDS plot, when the profile is reduced to 17 Y-STR loci, the phylogeny exhibited unexpected topologies or branch lengths among the reference populations potentially due to the bias of using less Y-STR markers. Among the Eastern Asian and European populations, the Uyghur population has a closer relationship with the Hui (Cangzhou, China), the Hungarian and the Mongolian populations. The proximity between the Uyghur population and the Hui population is consistent with historical records, which indicate that the present Hui population is an admixture of Central Asian, Han, Mongolian, Uyghur and other populations formed around the 13^th^ century. The relatively close relationship between the Uyghur population and the Hungarian population is consistent with the Asian origin hypothesis of Hungarians^33^,^34^,^35^,^36^,^37^. The proximity between the Uyghur population and the Mongolian population could be speculatively explained by the migration of Orkhon Uyghurs, proposed ancestors of present Uyghurs, from Mongolia to Xinjiang around the 9^th^ century. The migration allows gene flow between the Orkhon Uyghurs and the indigenes in Xinjiang, such as Tocharians, that are genetically similar to northern Europeans^28^,^38^,^39^. The fact that the indigenous population is much larger than the Orkhon Uyghur population may also explain why the Uyghur is genetically closer to European populations than Eastern Asian populations as is shown in this study.

### Forensic parameters of the 26 Y-STR loci system when applied to the Uyghur population

The allelic frequencies and GD values of the 26 Y-STR loci are shown in Table 3.Among the 26 Y-STR loci, DYS385ab and DYS388 exhibit the highest (0.8763) and the lowest (0.3665) GD values, respectively. The GD values of the Y-STR loci are greater than 0.5 with the exception of DYS388 (0.3665) and DYS391 (0.4972). The observed low genetic diversity at DYS391 is consistent with previous reports^39^,^40^,^41^.

A total of five variant alleles were observed in five samples when amplified using the Goldeneye^®^ system. An intermediate allele was observed at DYS449 (allele 34.1) in a single individual (Sample ID 98). In addition to DYS385ab, duplication variants were observed at DYS19 in one individual (Sample ID 9) and at DYS449 in another individual (Sample ID 35). Null alleles were observed at DYS448 in one individual (Sample ID 89), at DYS643 in one individual (Sample ID 5) and at Y_GATA_H4 in another individual (Sample ID 6). The high frequency of null allele at DYS448 was also observed in previous studies using other commercial kits^40^,^42^,^43^. Null alleles could arise due to either deletions within the target region (biological) or mutations within the primer binding sites (technical)^40^,^44^,^45^,^46^. Because the presence of null alleles could significantly affect statistics in population genetics^47^, it is important to distinguish biological null alleles from the null alleles caused by technical issues. To see if the null alleles are biological, AGCU Database Y24 and GFS 24Y STR kits with primers different from the Goldeneye^®^ system were used to amplify the markers exhibiting the null alleles. All samples showed the same results except for sample 6 at Y_GATA_H4, which exhibited allele type 11. The results suggested that biological null alleles and null alleles caused by technical issues could be, and should be, distinguished using different amplification systems. Intermediate alleles and duplication variants were also validated using AGCU Database Y24 and GFS 24Y kits; the results were not different from the Goldeneye^®^ system.

Multiplex Y-STR loci systems have extensive forensic applications including patrilineal relationship evaluation, mixture identification and ancestry inference. To evaluate the power of the 26 Y-STR loci system in forensic applications in Uyghur population, we measured forensic parameters of the 26 Y-STR loci system (Table 4). Out of the 100 Uyghur male samples typed in this study, 99 unique haplotypes were observed. The overall HD is 0.9998 with a DC of 0.9900. The results indicate that the 26 Y-STR loci system provides strong discriminatory power within the Uyghur population due to its high resolution. The system can be potentially used in population genetic studies and forensic practices because of its power to describe variation within the population.

We also compared the forensic parameters of the 26 Y-STR loci system to four different sets of Y-STR markers – the minimal 9 loci, PowerPlex Y12 loci, Y-filer 17 loci and PowerPlex Y23 loci – that are commonly used in forensic practices (Table 4). The 26 Y-STR loci system showed a significantly higher DC value and a significantly higher proportion of unique haplotypes (PUH) than the minimal 9 loci, PowerPlex Y12 loci and Y-filer 17 loci systems. The 26 Y-STR loci system had an equal discriminatory power with the PowerPlex Y23 system. It suggests that the introduction of more highly polymorphic Y-STR loci will likely increase the discriminatory power in forensic cases. Forensic usefulness of these multiplex Y-STR loci systems largely depends on the reference database that is being updated continuously as a global effort^48^,^49^,^50^. Nevertheless, there has been a lack of information for Uyghur samples in the database. Therefore haplotype data in this study would contribute to the Y-STR reference databases.

### The ability of the Y-STR loci to infer ancestry of DNA donors

Ancestry informative DNA markers are valuable tools in forensic sciences. For Uyghur samples, ancestry inference could be especially challenging due to the admixed nature of the population. We investigated the power of the Y-STR system in ancestry inference by asking how well it can discriminate Uyghur samples from different Asian and European samples.

**Figure 3:**
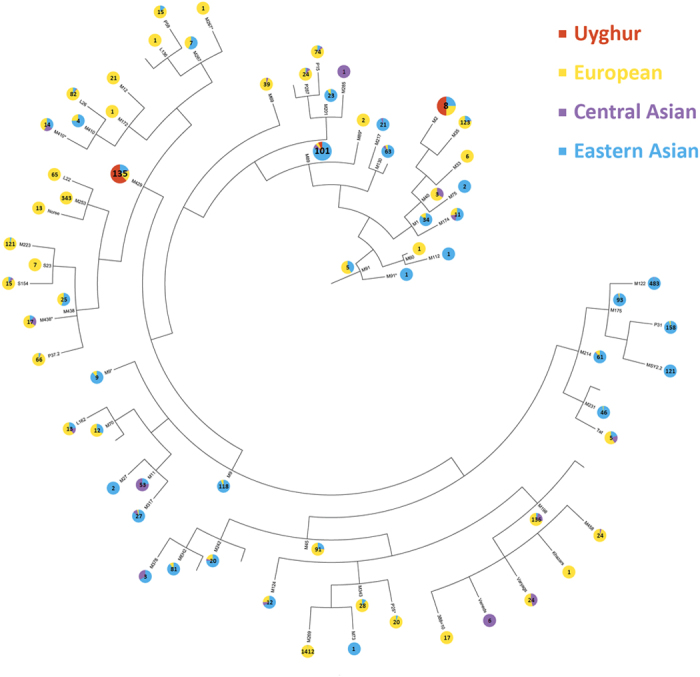
Y-DNA haplogroup tree of Uyghur, European, Central Asian and Eastern Asian samples. The number in each pie chart reflected the total number of samples in each haplogroup.

The Y-DNA haplogroup tree involving individual samples from Uyghur and reference populations revealed no clear separation of the Uyghur samples from the reference samples (Fig. 3). The Uyghur samples exhibited one primary haplogroup M429 containing 84 samples (out of 95 Uyghur samples used for the analysis) mixed with samples from mainly Eastern Asia and Europe, with the rest of the samples distributed in haplogroups M89 and M2. It is worthwhile to note that the relative abundance of Uyghur, Eastern Asian, Central Asian and European samples in each haplogroup also depends on the total number of samples used in the study that are from Uyghur, Eastern Asia, Central Asia and Europe.

**Figure 4:**
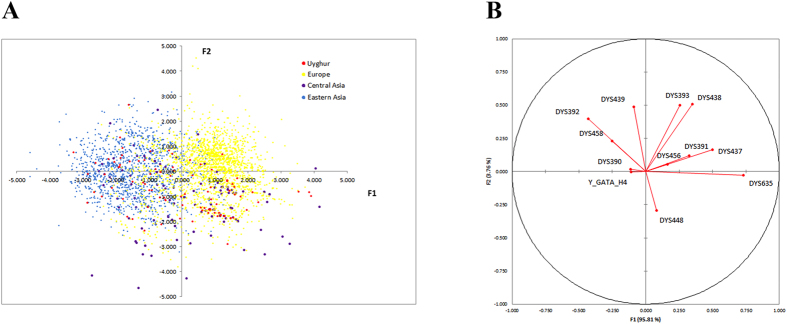
LDA of Uyghur, European, Central Asian and Eastern Asian samples based on Y-STR genotypes. (**A**) Uyghur, European, Central Asian and Eastern Asian samples plotted on the two LDA factors. (**B**) correlation coefficient of different Y-STR markers and the two LDA factors.

LDA was performed on the Uyghur, European, Central Asian and Eastern Asian samples to look for markers that are ancestry-informative. Figure 4A shows all individual samples plotted on the two LDA factors (axes F1 and F2). The first factor (F1) explained the majority (95.807%) of the variation. The markers DYS635 and DYS438 had the largest correlation coefficient (0.731 and 0.5) with the first and second factor, respectively (Fig. 4B). The plot showed no obvious separation of the Uyghur samples from the reference samples, although the Eastern Asian samples were well separated from the European samples. Due to the long history of admixture of Uyghurs, the present multiplex Y-STR data alone might be insufficient to discriminate Uyghur samples from European or Asian samples. A more comprehensive dataset that allows inclusion of more Y-STR loci may increase the power in finding ancestry-informative markers.

## Conclusions

In this study we genotyped 100 Uyghur males at 26 Y-STR loci and demonstrated that the 26 Y-STR loci system is useful in describing genetic variation in a Uyghur population in southern Xinjiang. Forensic parameters of the 26 Y-STR loci system showed that the system has high discriminatory power within the Uyghur population and has potential application in forensic studies. We showed that the Uyghur population from southern Xinjiang is genetically admixed with reference populations in Eastern Asia and Europe, with a slightly closer relationship to the European populations. Due to the admixed nature of Uyghur, it is hard to differentiate Uyghur DNA donors from donors in Asia or Europe based on the available Y-STR information.

## Additional Information

**How to cite this article**: Bian, Y. *et al.* Analysis of genetic admixture in Uyghur using the 26 Y-STR loci system. *Sci. Rep.* **6**, 19998; doi: 10.1038/srep19998 (2016).

## Supplementary Material

Supplementary Information

## Figures and Tables

**Table 1:** Reference Populations from Eastern Asia and Europe.

Country	Population	Sample size(haplotypes)
China	Bai (Yunnan)^48^*#	92
	Dai (Xishuangbanna)^48^*#	91
	Han (Beijing)^51^*#	238
	Han (Shanghai)^15^*#	516
	Han (Minnan)^52^*#	103
	Han (Chengdu)^48^^#^	99
	Han (Xuanwei)^48^^#^	145
	Hui (Cangzhou)*	346
	Manchu (Chengde)*	357
	Mongolian (Inner Mongolia)^53^*	160
	She (Fujian)^54^*	92
	Zhuang (Guangxi)^55^*#	371
South Korea	Korean^56^*#	1372
Japan	Japanese (Tokyo)^57^*#	153
Kazakhstan	Kazakh^20^*#	166
Afghanistan	Pathan^21^,^58^*#	268
Hungary	Hungarian^59^*#	303
	Romani^60^*#	291
Netherlands	Dutch^44^*#	2039
Spain	Spanish (Barcelona)^61^*#	76
United Kingdom	English (Southern England)^48^^#^	113
	English (Central England)^48^^#^	80
Ireland	Irish^62^*#	225

^*^populations selected as reference populations used in the analysis of molecular variance (AMOVA) and multidimensional scaling (MDS).

^#^popultions selected as reference populations used in Y-DNA haplogroup tree construction and linear discriminant analysis (LDA).

**Table 2:** Pairwise *R*_ST_ values.

Population	Uyghur	Bai	Dai	Han(BJ)	Han(MN)	Han(SH)	Hui	Manchu	Mongolian	She	Zhuang	Hungarian	Romani	Irish	Japanese	Korean	Dutch	Spanish	English(CE)	English(SE)
Uyghur	—	−1	−1	−1	−1	−1	−1	−1	−1	−1	−1	−1	−1	−1	−1	−1	−1	−1	−1	−1
Bai	0.098	—	−1	−1	−1	−1	−1	−1	−1	−1	−1	−1	−1	−1	−1	−1	−1	−1	−1	−1
Dai	0.1717	0.026	—	−1	−1	−1	−1	−1	−1	−1	−1	−1	−1	−1	−1	−1	−1	−1	−1	−1
Han(BJ)	0.069	0.0234	0.0378	—	−1	−1	−1	−1	−1	−1	−1	−1	−1	−1	−1	−1	−1	−1	−1	−1
Han(MN)	0.1437	0.043	0.0151	0.0182	—	−1	−1	−1	−1	−1	−1	−1	−1	−1	−1	−1	−1	−1	−1	−1
Han(SH)	0.0617	0.0318	0.0523	0.0026	0.0305	—	−1	−1	−1	−1	−1	−1	−1	−1	−1	−1	−1	−1	−1	−1
Hui	0.0132	0.0696	0.1212	0.0406	0.1018	0.0339	—	−1	−1	−1	−1	−1	−1	−1	−1	−1	−1	−1	−1	−1
Manchu	0.0618	0.0231	0.0392	−0.001	0.0258	0.0048	0.0351	—	−1	−1	−1	−1	−1	−1	−1	−1	−1	−1	−1	−1
Mongolian	0.0273	0.1134	0.1867	0.068	0.1372	0.0594	0.0378	0.0665	—	−1	−1	−1	−1	−1	−1	−1	−1	−1	−1	−1
She	0.1261	0.0658	0.0798	0.0278	0.0586	0.0459	0.0861	0.0308	0.1212	—	−1	−1	−1	−1	−1	−1	−1	−1	−1	−1
Zhuang	0.1402	0.0731	0.0588	0.0581	0.067	0.0594	0.1089	0.058	0.1586	0.1061	—	−1	−1	−1	−1	−1	−1	−1	−1	−1
Hungarian	0.0215	0.1865	0.258	0.1445	0.225	0.1315	0.0623	0.1357	0.065	0.2026	0.2038	—	−1	−1	−1	−1	−1	−1	−1	−1
Romani	0.0407	0.1423	0.2176	0.1036	0.1701	0.0976	0.0482	0.0969	0.0877	0.167	0.1634	0.0617	—	−1	−1	−1	−1	−1	−1	−1
Irish	0.1097	0.2418	0.3761	0.2329	0.3425	0.2196	0.1518	0.2162	0.1584	0.3185	0.3149	0.1284	0.2385	—	−1	−1	−1	−1	−1	−1
Japanese	0.1639	0.1131	0.1304	0.0951	0.1313	0.1073	0.1605	0.0911	0.1869	0.1202	0.1104	0.2299	0.2204	0.3329	—	−1	−1	−1	−1	−1
Korean	0.1592	0.0818	0.1016	0.0644	0.1001	0.0822	0.1374	0.061	0.1641	0.0869	0.1251	0.253	0.2299	0.3069	0.0602	—	−1	−1	−1	−1
Dutch	0.0663	0.2273	0.2996	0.2139	0.2919	0.1908	0.1132	0.1989	0.1239	0.2942	0.2805	0.0678	0.1753	0.0611	0.3359	0.2928	—	−1	−1	−1
Spanish	0.0553	0.1647	0.2785	0.1695	0.2641	0.1581	0.0856	0.1542	0.1106	0.254	0.2381	0.0844	0.1508	0.0259	0.2894	0.2545	0.0327	—	−1	−1
English(CE)	0.0675	0.2301	0.343	0.203	0.3141	0.1834	0.1136	0.1854	0.1193	0.2983	0.2828	0.069	0.1914	0.0219	0.3277	0.2876	0.0067	0.0224	—	−1
English(SE)	0.0605	0.2017	0.3065	0.1868	0.2876	0.1695	0.1023	0.1702	0.116	0.2719	0.2658	0.0722	0.1779	0.0266	0.3038	0.2641	0.003	0.0164	−0.0028	—

BJ, Beijing, China; MN, Minnan, China; SH, Shanghai; CE, Central England, United Kingdom; SE, Southern England, United Kingdom.

**Table 3:** Allelic frequency and gene diversity (GD) values for 26 Y-STR loci in the Uygur population from southern Xinjiang, China (n = 100).

Allele	DYS19	DYS389I	DYS389II	DYS390	DYS391	DYS392	DYS393	DYsS385ab	DYS437	DYS438	DYS439	DYS448	DYS456	DYS458	DYS635	Y_GATA_H4	DYS576	DYS570	DYS481	DYS533	DYS549	DYS643	DYS460	DYS449	DYS388
7						0.0500																			
8																						0.0400			
9					0.0300			0.0108		0.0800										0.0100		0.1300	0.1400		
10					0.6200	0.0200		0.0054		0.4100	0.3000					0.0600				0.0700	0.0100	0.4400	0.3500		0.0300
11					0.3500	0.4600	0.0100	0.2043		0.4000	0.2400					0.4200				0.3400	0.1000	0.2500	0.4600		
12		0.1600				0.0200	0.3400	0.1022		0.1000	0.3200					0.3400				0.4900	0.4800	0.1100	0.0500		0.7900
13	0.0990	0.4700				0.2300	0.5300	0.1505	0.0300	0.0100	0.1200		0.0100			0.1700	0.0100	0.0100		0.0800	0.2900	0.0100			0.0600
14	0.2673	0.3600				0.1700	0.1000	0.1882	0.5700		0.0200		0.1300	0.0400		0.0100	0.0100	0.0200		0.0100	0.1000	0.0100			0.0900
15	0.4356	0.0100				0.0300	0.0200	0.0860	0.3300				0.5500	0.2800			0.0200	0.0100			0.0200				0.0200
16	0.1089					0.0200		0.0860	0.0700			0.0100	0.2200	0.2000			0.1200	0.1400							0.0100
17	0.0891							0.0699					0.0700	0.1900			0.2700	0.2400							
18								0.0323				0.0800	0.0200	0.1700			0.3300	0.2100							
19				0.0300				0.0108				0.4200		0.0500	0.0500		0.1700	0.2200	0.0100						
20				0.0100				0.0323				0.3700		0.0600	0.0700		0.0600	0.1100	0.0400						
21				0.0000				0.0108				0.0900		0.0100	0.2900		0.0100	0.0300	0.0500						
22				0.0700				0.0054				0.0200			0.2000				0.1200						
23				0.3200											0.2800				0.2600						
24				0.2900				0.0054							0.0900			0.0100	0.2100						
25				0.2500															0.2100					0.0099	
26				0.0300											0.0100				0.0600					0.0198	
27			0.0200												0.0100				0.0300					0.0495	
28			0.1000																0.0100					0.0297	
29			0.2800																					0.1089	
30			0.3300																					0.1485	
31			0.1800																					0.1980	
32			0.0900																					0.2673	
33																								0.1089	
34																								0.0297	
34.1																								0.0099	
35																								0.0198	
null												0.0100										0.0100			
GD	0.7163	0.6301	0.7695	0.7517	0.4972	0.7091	0.5990	0.8763	0.5661	0.6620	0.7426	0.6784	0.6331	0.8170	0.7897	0.6822	0.7784	0.8248	0.8293	0.6392	0.6717	0.7202	0.6503	0.8472	0.3665

**Table 4:** Forensic statistical parameters.

Haplotypes	Minimal 9 loci	PowerPlex Y12 loci	Y-filer 17 loci	PowerPlex Y23 loci	26-Y loci
Sample size	100	100	100	100	100
Number of haplotypes	89	92	97	99	99
Unique haplotypes	81	85	95	98	98
PUH	0.8100	0.8500	0.9500	0.9800	0.9800
MP	0.0128	0.0118	0.0108	0.0102	0.0102
DC	0.8900	0.9200	0.9700	0.9900	0.9900
HD ± SE	0.9972 ± 0.0004	0.9982 ± 0.0003	0.9992 ± 0.0002	0.9998 ± 0.0001	0.9998 ± 0.0001

PUH, proportion of unique haplotypes; MP, match probability; DC, discriminatory capacity; HD, haplotype diversity; SE, standard error.
