# Supplementary material for: Analysis of genetic admixture in Uyghur using the 26 Y-STR loci system
Source: Sci Rep. 2016 Feb 4;6:19998. doi: 10.1038/srep19998 (PMC4740765; doi:10.1038/srep19998)
Supplement: Supplementary Information [file srep19998-s1.pdf]

Supplementary Table S1 and Supplementary Figure S1

Analysis of genetic admixture in Uyghur using the 26 Y-STR loci system

Yingnan Bian[1a], Shuhua Zhang[1,2a], Wei Zhou[3a], Siqintuya[4], Zhao Qi[1,5], Ruxin Zhu[1], Zheng Wang[1], Yuzhen Gao[5], Jie Hong[6], Daru Lu[2], Chengtao Li[1*]

1. Shanghai Key Laboratory of Forensic Medicine, Institute of Forensic Sciences, Ministry of Justice, P.R. China, Shanghai 200063, China

2. State Key Laboratory of Genetic Engineering, Institute of Genetics, School of Life Sciences, Fudan University, Shanghai 200433, China

3. University of Pennsylvania, 3451 Walnut Street, Philadelphia, PA 19104, United States

4. Shihezi City Public Security Bureau Traffic Institute of Science and Technology, Xinjiang 832000, China

4. State Key Laboratory of Genetic Engineering, Institute of Genetics, School of Life Science, Fudan University, Shanghai 200433, China

5. Department of Forensic Medicine, Medical College of Soochow University, Suzhou 215123, China

6. Division of Gastroenterology and Hepatology, Renji Hospital, Shanghai Institution of Digestive Disease; Key Laboratory of Gastroenterology and Hepatology, Ministry of Health; State Key Laboratory of Oncogene and Related Genes, Shanghai Jiao-Tong University School of Medicine, Shanghai 200001, China

a. These authors contributed equally to this work.

* Correspondence and requests for materials should be addressed to Chengtao Li (lichengtaohla@163.com)

Supplementary Table S1. Haplotypes for 26 Y-STR loci detected in 100 unrelated males of Uygur from southern Xingjiang, China.

ID	Population	DYS19	DYS389I	DYS389II	DYS390	DYS391	DYS392	DYS393	DYS385ab	DYS437	DYS438	DYS439	DYS448	DYS456	DYS458	DYS635	Y_GATA_H4	DYS576	DYS570	DYS481	DYS533	DYS549	DYS643	DYS460	DYS449	DYS388
1	Xinjiang, China[Uyghur]	13	12	30	25	11	13	14	13,20	15	10	11	19	15	16	22	13	19	17	23	11	14	9	9	27	12
2	Xinjiang, China[Uyghur]	15	12	28	25	11	11	13	11,14	14	11	10	20	14	15	23	13	18	17	23	12	12	10	11	33	12
3	Xinjiang, China[Uyghur]	15	13	29	24	10	11	13	12,14	14	11	12	16	17	17	21	11	17	20	27	11	12	10	9	25	13
4	Xinjiang, China[Uyghur]	14	14	31	23	11	14	12	11,12	14	10	10	18	14	18	20	11	17	18	21	12	12	12	10	32	12
5	Xinjiang, China[Uyghur]	14	12	28	24	10	11	12	14,17	15	9	11	21	15	16	21	11	18	16	24	13	12		11	32	15
6	Xinjiang, China[Uyghur]	17	12	28	24	10	13	12	14,19	15	10	13	20	15	21	21	11	18	20	26	11	12	11	10	31	12
7	Xinjiang, China[Uyghur]	17	12	27	23	10	13	12	12,17	15	10	12	19	14	18	21	11	18	16	24	12	14	12	9	30	12
8	Xinjiang, China[Uyghur]	14	13	29	24	10	14	12	11,14	15	12	12	18	15	14	23	12	18	13	22	11	13	10	10	29	12
9	Xinjiang, China[Uyghur]	16,17	14	31	23	9	11	13	12	14	10	11	20	15	18	24	10	19	17	27	11	12	9	11	32	12
10	Xinjiang, China[Uyghur]	16	14	30	24	10	11	13	11,14	14	11	10	20	15	16	23	13	18	21	24	12	12	10	11	31	12
11	Xinjiang, China[Uyghur]	15	12	29	23	10	13	12	12,17	15	10	11	19	15	16	19	12	19	17	22	11	14	12	9	32	12
12	Xinjiang, China[Uyghur]	13	14	30	24	9	14	14	15,22	14	12	10	19	15	15	22	10	19	18	23	12	12	10	10	31	12
13	Xinjiang, China[Uyghur]	14	13	29	24	10	11	12	13,16	15	10	11	19	15	20	21	11	17	18	23	12	13	9	11	30	14
14	Xinjiang, China[Uyghur]	16	13	31	24	10	13	12	13,17	15	9	10	19	15	16	21	13	18	14	25	12	15	9	9	32	12
15	Xinjiang, China[Uyghur]	15	12	29	23	10	11	14	13,14	16	10	11	20	17	17	21	12	16	17	21	9	12	11	10	29	12
16	Xinjiang, China[Uyghur]	15	14	32	25	10	11	13	11,14	14	11	10	20	16	15	23	13	18	19	24	12	13	10	11	31	12
17	Xinjiang, China[Uyghur]	15	14	30	25	11	13	12	12,17	15	10	12	19	14	17	24	14	19	19	24	11	12	10	10	31	12
18	Xinjiang, China[Uyghur]	14	14	30	19	11	13	13	13	15	10	13	19	15	18	24	11	16	17	24	13	12	8	10	32	12
19	Xinjiang, China[Uyghur]	15	13	32	25	10	11	13	11,14	14	11	12	20	16	15	23	12	18	18	25	11	12	10	11	33	12
20	Xinjiang, China[Uyghur]	14	13	31	23	10	14	14	11,13	14	10	10	19	15	18	22	12	18	20	20	11	11	11	10	30	12
21	Xinjiang, China[Uyghur]	15	14	30	25	11	11	13	11,13	14	11	10	20	16	14	23	12	17	19	23	12	12	10	10	34	12
22	Xinjiang, China[Uyghur]	13	13	32	25	11	11	13	15,17	14	10	13	20	16	19	22	11	16	18	25	10	12	11	10	31	12
23	Xinjiang, China[Uyghur]	15	13	30	25	11	11	13	11,15	14	11	10	20	15	15	23	12	21	17	23	12	13	10	11	33	12
24	Xinjiang, China[Uyghur]	16	13	30	24	11	11	13	11,20	14	11	10	20	14	15	23	13	16	19	23	12	12	10	12	30	12
25	Xinjiang, China[Uyghur]	14	13	28	25	10	13	12	13,20	15	11	12	20	15	18	20	12	18	18	23	11	13	12	9	32	10
26	Xinjiang, China[Uyghur]	15	12	29	23	10	12	13	12,17	14	10	12	18	16	18	19	13	19	16	22	13	13	11	9	32	12
27	Xinjiang, China[Uyghur]	16	13	28	23	10	14	12	13,17	14	10	12	20	15	17	21	11	17	17	25	11	12	10	10	31	12
28	Xinjiang, China[Uyghur]	14	14	30	23	10	15	14	11,13	14	10	10	19	13	17	21	13	16	19	20	10	11	11	11	29	12
29	Xinjiang, China[Uyghur]	15	13	30	25	10	11	13	12,14	14	11	10	20	15	16	23	11	19	20	23	12	13	10	11	32	12
30	Xinjiang, China[Uyghur]	15	14	30	25	10	7	13	11	14	11	13	19	15	17	21	11	18	19	26	12	14	11	10	32	12
31	Xinjiang, China[Uyghur]	13	13	31	24	10	14	13	14,17	14	11	12	18	15	18	22	11	19	17	24	12	13	10	10	29	12
32	Xinjiang, China[Uyghur]	16	13	29	23	10	11	15	11,18	14	10	11	21	15	16	22	11	17	17	22	14	13	9	11	30	14
33	Xinjiang, China[Uyghur]	15	13	30	24	9	11	13	12	14	10	11	20	14	19	23	10	19	17	27	11	12	9	11	32	13
34	Xinjiang, China[Uyghur]	14	13	29	24	11	13	12	11,15	15	12	13	19	15	18	23	12	17	18	22	12	13	10	10	28	12
35	Xinjiang, China[Uyghur]	14	13	29	23	11	13	12	12,13	14	12	12	19	16	15	23	13	17	17	21	12	12	10	11	29,30	12
36	Xinjiang, China[Uyghur]	14	14	30	24	10	11	12	13,16	15	10	11	19	15	19	21	11	17	19	23	12	14	9	11	29	14
37	Xinjiang, China[Uyghur]	15	13	29	23	10	11	15	13,17	14	10	11	21	15	16	21	11	17	17	26	12	13	8	10	32	13
38	Xinjiang, China[Uyghur]	14	13	30	25	10	14	12	13,21	15	10	12	19	15	18	23	11	14	17	24	10	13	10	9	33	12
39	Xinjiang, China[Uyghur]	14	13	29	23	10	11	12	13,16	15	10	11	19	15	20	21	11	17	18	23	12	14	9	11	29	14
40	Xinjiang, China[Uyghur]	15	12	28	23	11	12	12	12,16	15	10	12	19	16	20	20	12	18	17	22	11	13	11	10	33	12
41	Xinjiang, China[Uyghur]	15	13	30	25	11	11	13	11,14	14	11	10	20	17	15	23	12	17	20	23	12	11	10	11	32	12
42	Xinjiang, China[Uyghur]	14	14	29	19	11	13	13	13	15	10	12	19	15	17	24	11	16	17	24	13	12	8	10	33	12
43	Xinjiang, China[Uyghur]	17	12	28	24	10	13	12	14,20	15	10	12	20	15	18	22	11	18	19	26	12	12	11	10	32	12
44	Xinjiang, China[Uyghur]	16	14	30	24	10	14	13	11,13	14	11	11	19	17	15	24	12	17	20	25	13	13	13	10	30	12
45	Xinjiang, China[Uyghur]	15	13	29	22	10	14	12	9,16	16	10	13	20	15	17	22	11	15	15	24	12	11	11	11	31	12
46	Xinjiang, China[Uyghur]	15	14	30	23	11	11	13	14,16	16	10	11	22	15	18	23	12	17	16	19	11	12	12	11	27	12
47	Xinjiang, China[Uyghur]	15	13	31	25	11	11	13	11,15	14	11	10	20	16	15	23	13	18	19	23	11	12	10	12	32	12
48	Xinjiang, China[Uyghur]	13	14	31	23	11	16	13	14,15	14	11	12	21	15	18	22	11	17	20	25	11	11	10	11	26	12
49	Xinjiang, China[Uyghur]	15	14	31	23	10	11	13	14,17	16	10	12	21	18	16	21	10	16	18	20	10	12	12	11	28	12
50	Xinjiang, China[Uyghur]	15	13	29	22	11	15	12	9,16	16	10	12	19	15	19	22	12	17	16	24	12	12	11	10	29	12
51	Xinjiang, China[Uyghur]	16	12	27	22	11	11	12	12,16	15	9	12	20	15	15	21	12	15	16	22	11	13	11	10	31	15
52	Xinjiang, China[Uyghur]	15	14	29	23	10	11	14	11,18	14	10	12	21	15	17	20	11	18	16	25	12	12	9	10	29	12
53	Xinjiang, China[Uyghur]	15	14	32	26	11	11	13	11,15	14	11	10	20	16	16	23	13	18	18	24	12	13	10	11	33	12
54	Xinjiang, China[Uyghur]	15	14	31	25	10	11	13	11,14	14	11	10	20	15	16	23	12	20	16	24	12	12	10	11	32	12
55	Xinjiang, China[Uyghur]	15	13	29	26	10	11	13	11,15	14	11	11	20	18	15	23	12	17	19	24	12	10	10	11	31	12
56	Xinjiang, China[Uyghur]	15	13	29	24	10	13	12	12,20	15	11	13	20	15	15	21	11	18	18	24	11	12	11	9	33	10
57	Xinjiang, China[Uyghur]	15	14	30	23	10	14	13	13	14	10	11	18	17	15	19	12	17	20	25	11	12	11	10	31	12
58	Xinjiang, China[Uyghur]	13	13	30	23	10	14	13	15,16	13	12	14	19	15	16	22	11	20	19	25	11	11	11	10	30	12
59	Xinjiang, China[Uyghur]	14	12	28	23	10	11	13	14	16	10	11	21	14	15	22	11	18	21	25	12	13	12	10	28	14

Supplementary Table S1. Haplotypes for 26 Y-STR loci detected in 100 unrelated males of Uygur from southern Xingjiang, China. (Continued)

ID	Population	DYS19	DYS389I	DYS389II	DYS390	DYS391	DYS392	DYS393	DYS385ab	DYS437	DYS438	DYS439	DYS448	DYS456	DYS458	DYS635	Y_GATA_H4	DYS576	DYS570	DYS481	DYS533	DYS549	DYS643	DYS460	DYS449	DYS388
60	Xinjiang, China[Uyghur]	13	14	30	22	10	15	13	11,18	14	11	14	18	15	17	22	10	20	16	25	10	14	12	11	27	12
61	Xinjiang, China[Uyghur]	17	14	31	25	11	11	13	11,14	14	11	11	20	15	16	23	12	18	18	23	13	12	10	11	31	12
62	Xinjiang, China[Uyghur]	17	13	31	25	10	11	14	11,14	14	11	10	20	15	14	23	13	19	19	25	12	12	10	11	33	12
63	Xinjiang, China[Uyghur]	14	13	29	22	11	14	11	13,17	15	11	13	19	16	15	22	12	16	16	23	12	12	10	10	27	13
64	Xinjiang, China[Uyghur]	15	14	30	25	10	7	13	11	14	11	12	19	15	17	21	11	19	19	26	12	13	11	11	32	12
65	Xinjiang, China[Uyghur]	15	13	32	24	10	11	13	11,14	14	11	10	20	16	15	23	12	17	19	23	12	12	10	11	32	12
66	Xinjiang, China[Uyghur]	14	13	30	20	10	13	13	12,14	15	12	12	19	15	14	27	11	18	17	23	13	12	11	11	32	12
67	Xinjiang, China[Uyghur]	15	14	31	25	10	11	13	11,14	14	11	10	20	16	16	24	12	18	21	24	12	12	10	11	33	12
68	Xinjiang, China[Uyghur]	15	12	29	23	10	13	12	12,17	14	10	12	19	16	16	19	11	18	18	24	11	13	11	9	34	12
69	Xinjiang, China[Uyghur]	14	12	29	24	11	11	12	14,15	14	9	12	19	14	16	22	12	18	17	23	11	12	10	11	29	14
70	Xinjiang, China[Uyghur]	17	13	31	24	11	11	13	11,14	14	11	10	20	15	17	23	13	16	19	24	12	13	10	11	30	12
71	Xinjiang, China[Uyghur]	15	14	29	23	10	10	14	13,16	15	11	10	19	14	17	26	12	19	16	25	11	13	10	10	32	12
72	Xinjiang, China[Uyghur]	15	14	32	24	11	11	13	11,15	14	11	10	20	15	15	23	13	19	19	23	12	12	10	11	30	12
73	Xinjiang, China[Uyghur]	15	12	29	23	10	13	12	13,18	15	10	12	19	15	17	19	12	18	17	22	10	12	14	9	35	12
74	Xinjiang, China[Uyghur]	14	13	29	24	10	11	12	13,16	15	10	11	19	15	20	21	11	18	19	23	12	13	9	11	31	14
75	Xinjiang, China[Uyghur]	15	14	31	25	10	7	13	11	14	11	12	19	15	17	21	11	18	19	26	11	13	11	11	31	12
76	Xinjiang, China[Uyghur]	15	13	30	26	11	11	13	11	14	11	10	20	16	15	24	13	18	18	23	12	12	10	12	35	12
77	Xinjiang, China[Uyghur]	14	13	31	23	10	14	14	11,13	14	10	10	19	14	17	22	12	17	20	20	12	11	11	11	30	12
78	Xinjiang, China[Uyghur]	14	14	31	23	10	11	12	14,19	15	9	12	20	16	15	21	12	18	16	22	11	11	10	11	31	16
79	Xinjiang, China[Uyghur]	15	13	32	24	11	11	13	11,14	14	11	10	20	15	15	23	12	17	20	23	12	12	10	11	34	12
80	Xinjiang, China[Uyghur]	16	13	30	24	11	11	12	11,15	14	11	10	20	16	15	23	12	18	19	22	12	12	10	12	32	12
81	Xinjiang, China[Uyghur]	15	13	30	24	10	13	12	13,18	15	9	10	19	15	15	21	13	19	14	25	12	15	9	9	32	12
82	Xinjiang, China[Uyghur]	13	15	32	23	11	16	13	14,15	14	11	12	21	16	18	22	11	17	19	25	11	12	10	11	26	12
83	Xinjiang, China[Uyghur]	15	14	30	19	11	13	13	13,14	15	10	13	19	15	17	24	11	16	17	24	12	12	8	10	33	12
84	Xinjiang, China[Uyghur]	14	13	29	25	11	13	12	14	15	12	13	19	16	16	23	12	17	16	23	12	13	9	11	31	12
85	Xinjiang, China[Uyghur]	14	14	30	23	11	10	13	14,18	16	11	11	19	15	15	24	13	16	17	25	10	13	10	12	32	12
86	Xinjiang, China[Uyghur]	16	14	31	25	11	11	13	11,14	14	12	10	21	16	15	23	10	18	17	24	13	12	10	11	32	12
87	Xinjiang, China[Uyghur]	16	14	30	25	10	7	13	11	14	11	12	19	16	16	21	11	19	18	25	11	13	11	11	30	12
88	Xinjiang, China[Uyghur]	14	14	32	23	10	13	13	14,16	14	9	11	19	17	17	21	11	16	17	22	12	14	10	10	31	12
89	Xinjiang, China[Uyghur]	15	13	29	24	10	11	13	12,15	14	11	12		14	18	21	11	17	24	28	12	12	10	9	29	13
90	Xinjiang, China[Uyghur]	13	13	30	23	10	14	13	15,16	13	12	13	19	15	16	22	12	20	19	25	11	11	11	10	30	12
91	Xinjiang, China[Uyghur]	15	13	29	22	10	13	14	10,21	14	13	12	18	14	19	20	12	19	17	22	11	12	12	11	32	12
92	Xinjiang, China[Uyghur]	14	14	31	23	11	14	12	11,12	14	10	10	18	14	18	20	11	17	18	21	12	12	12	10	32	12
93	Xinjiang, China[Uyghur]	13	13	30	23	10	14	13	15,16	13	12	13	19	15	15	22	11	20	18	25	11	11	11	10	30	12
94	Xinjiang, China[Uyghur]	17	12	28	24	10	13	13	14,20	15	10	12	20	15	17	21	12	18	18	21	11	13	11	11	32	13
95	Xinjiang, China[Uyghur]	15	14	30	25	10	7	13	11	14	11	12	19	16	16	22	11	18	20	25	11	14	10	11	31	12
96	Xinjiang, China[Uyghur]	17	13	29	25	10	11	13	12	14	10	10	22	15	18	21	11	17	16	25	12	12	10	10	27	14
97	Xinjiang, China[Uyghur]	15	13	30	24	11	13	13	13,24	15	10	11	20	15	15	20	11	17	19	24	11	13	12	9	31	12
98	Xinjiang, China[Uyghur]	14	13	28	24	10	14	12	12,13	15	11	11	20	16	15	21	12	19	18	23	11	13	11	10	34.1	10
99	Xinjiang, China[Uyghur]	15	14	30	22	10	11	12	14,16	14	9	11	19	17	20	21	11	13	18	23	12	12	9	11	31	12
100	Xinjiang, China[Uyghur]	14	13	29	24	10	11	12	13,16	15	10	11	19	15	20	21	11	20	18	23	12	14	10	10	30	14

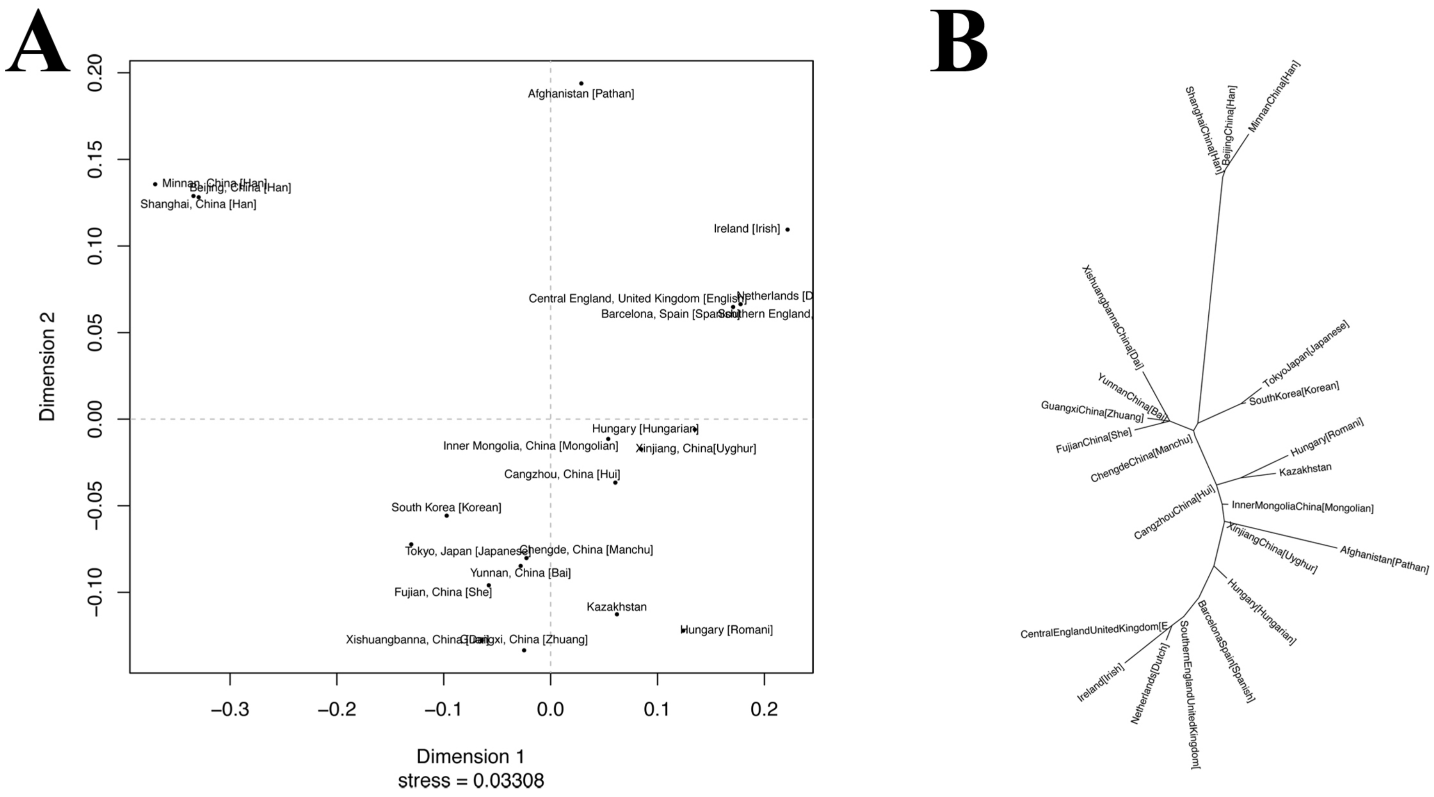

Supplementary Figure S1. MDS plot and neighbour-joining phylogenetic tree. A, MDS plot of the Uyghur population and 21 different European, Central Asian or Eastern Asian populations based on R_{ST}. B, Neighbour-joining phylogenetic tree of the Uyghur population and the European, Central Asian and Eastern Asian populations based on a distance matrix of R_{ST}.
